# Optimal timing of precut sphincterotomy to prevent post‐endoscopic retrograde cholangiopancreatography pancreatitis in difficult biliary cannulation: A retrospective study

**DOI:** 10.1002/deo2.70138

**Published:** 2025-05-06

**Authors:** Tomohiro Tanikawa, Keisuke Miyake, Mayuko Kawada, Katsunori Ishii, Takashi Fushimi, Noriyo Urata, Nozomu Wada, Ken Nishino, Mitsuhiko Suehiro, Miwa Kawanaka, Hidenori Shiraha, Ken Haruma, Hirofumi Kawamoto

**Affiliations:** ^1^ Department of General Internal Medicine 2 Kawasaki Medical School Okayama Japan

**Keywords:** biliary tract diseases, ERCP, pancreatic stent, post‐ERCP pancreatitis, precut papillotomy

## Abstract

**Objectives:**

Precut sphincterotomy is often performed when bile duct cannulation is difficult; however, the former has a higher risk of complications than conventional methods. Early precut reduces the risk of post‐endoscopic retrograde cholangiopancreatography pancreatitis (PEP). This study aimed to determine the appropriate timing for precut sphincterotomy to minimize the incidence of PEP.

**Methods:**

This retrospective study analyzed 320 patients who underwent precut sphincterotomy during their first endoscopic retrograde cholangiopancreatography at a single center. The optimal precut timing was identified using receiver operating characteristic analysis. Patients were divided into an optimized precut group (≤12 min, *n* = 198) and a delayed group (>12 min, *n* = 122). The incidence and risk factors of PEP were evaluated using multivariate analyses.

**Results:**

Receiver operating characteristic analysis identified 12.5 min as the optimal cutoff for transitioning to precut sphincterotomy (area under the curve, 0.613; sensitivity, 61.5%; specificity, 63.9%). The incidence of PEP was significantly lower in the optimized precut group than in the delayed precut group (5.1% vs. 13.1%, *p* = 0.02). Multivariate analysis identified delayed precut timing (odds ratio [OR], 3.134; *p* = 0.04) and the absence of endoscopic pancreatic stenting (OR, 0.284; *p* = 0.01) as independent risk factors for PEP.

**Conclusion:**

Precut sphincterotomy within 12.5 min of a cannulation attempt reduces the risk of PEP while maintaining procedural safety. Additionally, endoscopic pancreatic stenting can reduce PEP, even in precut scenarios.

## INTRODUCTION

Endoscopic retrograde cholangiopancreatography (ERCP) is important for diagnosing and treating biliary diseases, and deep cannulation is the first and the most critical step in ERCP. Success directly affects the effectiveness of the procedure and patient prognosis. Although the success rate of deep biliary cannulation was reported to be 85%–95%,^1^, ^2^ approximately 5–10% of the attempts have been reported as difficult cannulation cases that required additional techniques to achieve biliary access. Various techniques such as precut sphincterotomy, pancreatic guidewire‐assisted methods, and rendezvous techniques have been used to achieve deep biliary cannulation.^3^

However, the procedure is associated with severe complications, and post‐ERCP pancreatitis (PEP) is one of the most common complications of ERCP. The incidence of PEP ranges from 2.1% to 10.2%,^4^, ^5^, ^6^ and it can lead to severe outcomes. The risk factors for PEP can be categorized into patient‐ and procedure‐related. Patient‐related factors include younger age, female sex, and sphincter of Oddi dysfunction. Procedural factors include difficult bile duct cannulation and inadvertent pancreatic duct cannulation.^6^, ^7^ Precut sphincterotomy has also been reported as a risk factor for PEP.^8^, ^9^, ^10^, ^11^ However, some recent studies have suggested that early precut could reduce the incidence of PEP.^12^, ^13^ Our previous study also reported that precut was not a risk factor for PEP and that early precutting contributed to PEP prevention.^14^ Except for PEP, precut sphincterotomy also poses risks for other complications, such as bleeding and perforation. Therefore, their implementation must be carefully considered.

However, despite some reports stating that early precut has the potential for PEP prevention, there is no consensus on the optimal timing to start precut sphincterotomy. To resolve this issue, we conducted a retrospective study to determine the appropriate timing for initiating precut sphincterotomy in patients with difficult bile duct cannulation.

## METHODS

This retrospective observational study adhered to the principles of the 1975 Declaration of Helsinki and was approved by the Institutional Research Ethics Committee (Approval No: 5984‐01).

### Patients

**FIGURE 1:**
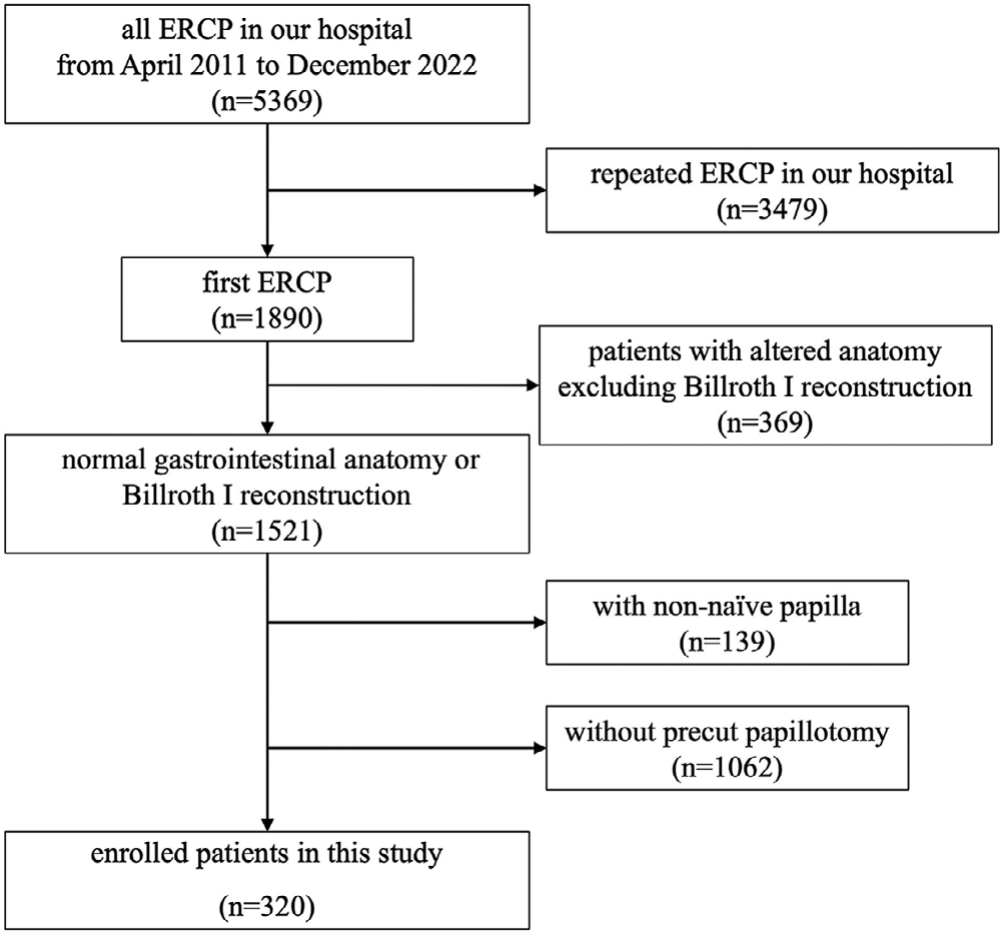
The flow diagram of patient selection of this study 164 × 155 mm (300 × 300 DPI).

The patient selection process is summarized in Figure 1. This retrospective study was conducted at a single center, Kawasaki University General Medical Center, from April 2011 to December 2022. We enrolled 320 patients aged ≥20 years with normal gastrointestinal anatomy and Billroth I reconstruction with naïve papillae, who underwent precut sphincterotomy during their first ERCP attempt for biliary disease. The exclusion criteria were indications for a pancreatic procedure or pregnancy and not being allowed to provide informed consent. The included patients included 169 males (52.8%), with a mean age of 77.0 ± 13.7 years.

### Procedure

A side‐viewing endoscope (JF‐260 V or TJF‐260V; Olympus Co. Ltd.) was used, and endoscopic retrograde cholangiography was initially performed at our institution using the conventional contrast method. When the operator was a trainee, deep bile duct cannulation was allowed for up to 15 min; if unsuccessful, the trainer took over. In cases where the pancreatic duct was cannulated during the trainee's attempt, the pancreatic duct guidewire method might have been employed; however, precut sphincterotomy was not performed. When the trainer took over, the participants began using the contrast method. If deep biliary cannulation remained difficult, precut sphincterotomy was performed without time restrictions. In cases in which access to the papilla is feasible, a precut sphincterotomy is generally preferred as the next step after the contrast method.

When the trainer was the first operator, the procedure was initiated using a conventional contrast method. If deep cannulation was difficult, we performed a precut sphincterotomy without a specific time limit. At our institution, precut sphincterotomy is typically the second conventional contrast cannulation method. This is because of its applicability to various cases, including those in which pancreatic duct cannulation is not achieved, and its advantage of minimizing pancreatic duct stimulation, making it a valuable approach. At our institution, a trainer was defined as an endoscopist who performed >1000 ERCP.

Precut sphincterotomy techniques include needle‐knife precut papillotomy (NKPP), needle‐knife fistulotomy, trans‐pancreatic sphincterotomy, or needle‐knife sphincterotomy with pancreatic stenting. We mainly used NKPP with a KD‐10Q‐1 (Olympus Co. Ltd.) because of its wide adaptability and precision in incising the sphincters.

In cases with difficult biliary cannulation or residual contrast medium in the pancreatic duct after the procedure, a temporary pancreatic stent was placed to reduce the risk of PEP. At the end of the ERCP procedure, a guidewire was inserted into the pancreatic duct and a temporary plastic pancreatic stent was placed. Either a 4Fr 3 cm Advanix stent (Boston Scientific) or a 5Fr, 3 cm Geenen stent (Cook Medical) was used. Rectal diclofenac was not administered to any patients included in this study.

### Statistical analysis

Continuous variables were presented as mean ± standard deviation and compared using the Mann–Whitney U test. Categorical variables were reported as frequencies (percentages) and compared using the chi‐square test. The clinical characteristics were compared between the non‐PEP and PEP groups. Receiver operating characteristic (ROC) curves were constructed to identify the optimal timing for precut sphincterotomy. Based on the results of the ROC analysis, precut cases were divided into two groups according to the optimal cutoff value: the optimized and delayed precut groups. The incidence of PEP was compared between the two groups using the chi‐square test. Subsequently, binary logistic regression analysis was performed to identify independent risk factors for PEP. Variables included in the binary logistic regression model were precut timing, age, sex, operator experience, total procedure time, endoscopic pancreatic stenting (EPS), cannulation time, and diverticula. Variables included in the binary logistic regression model were precut timing, age, sex, operator experience, total procedure time, EPS, cannulation time, and presence of diverticula. These variables were selected not only based on their statistical significance in univariate analysis but also based on clinical relevance and findings from previous literature.^5^, ^9^ This approach was chosen to ensure comprehensive adjustment for potential confounders, even if some variables were not statistically significant in the univariate analysis. Adjusted odds ratios (ORs) and 95% confidence intervals (CIs) were calculated to quantify the association between the potential risk factors and PEP occurrence. All statistical analyses were performed using SPSS version 29 (IBM Corp.). Statistical significance was set at *p* < 0.05 for all analyses.

In this study, PEP was diagnosed based on the Revised Atlanta Classification, which requires the presence of at least two of the following three features: (1) abdominal pain consistent with acute pancreatitis; (2) serum lipase or amylase activity at least three times the upper limit of normal; and (3) characteristic findings of acute pancreatitis on computed tomography. PEP severity was assessed using the Cotton classification, which categorizes cases as mild (2–3 days of hospitalization), moderate (4–10 days of hospitalization), or severe (≥10 days of hospitalization).

## RESULTS

### Patient characteristics

A total of 320 patients who underwent a precut papillotomy during the first ERCP were included in the analysis. The mean age of the patients was 77.0 ± 13.7 years, and 52.8% were male. Among the included patients, PEP occurred in 48 (15%), and the clinical characteristics of the PEP and non‐PEP groups are summarized in Table 1.

**TABLE 1:** Characteristics of included patients.

	PEP (‐) *n* = 294	PEP (+) *n* = 26	*p*‐value
Age	77.0 ± 13.9	74.0 ± 10.2	0.61
Gender, male	154 (52.4%)	15 (57.7%)	0.68
Anatomy			0.11
Normal	282 (95.9%)	23 (88.5%)	
Billroth I	12 (4.1%)	3 (11.5%)	
Diagnosis			1.00
Benign	201 (68.4%)	18 (69.2%)	
Malignancy	93 (31.6%)	8 (30.8%)	
First endoscopist			0.31
Trainee	145 (49.3%)	16 (61.5%)	
Trainer	149 (50.7%)	10 (38.5%)	
Diverticular	75 (25.5%)	7 (26.9%)	0.82
Precut start time	10.0 ± 7.5	13.0 ± 8.9	0.05
Cannulation time	17.0 ± 12.5	18.5 ± 15.5	0.48
Success of cannulation	280 (95.2%)	24 (92.3%)	0.38
Additional endoscopic papillary procedures (not mutually exclusive)			
Non	122 (41.5%)	15 (57.7%)	0.082
EST	141 (48.0%)	8 (30.8%)	0.07
EPBD	28 (9.5%)	1 (3.8%)	0.60
EPLBD	20 (6.8%)	2 (7.7%)	0.94
EPS	144 (49.0%)	6 (23.1%)	0.01
Total procedure time	38.0 ± 15.5	39.5 ± 18.2	0.67

Abbreviations: EPBD, endoscopic papillary balloon dilation; EPLBD, endoscopic papillary large balloon dilation; EPS, endoscopic pancreatic stenting; EST, endoscopic sphincterotomy; PEP, post‐endoscopic retrograde cholangiopancreatography pancreatitis.

### Optimal timing for precut papillotomy

**FIGURE 2:**
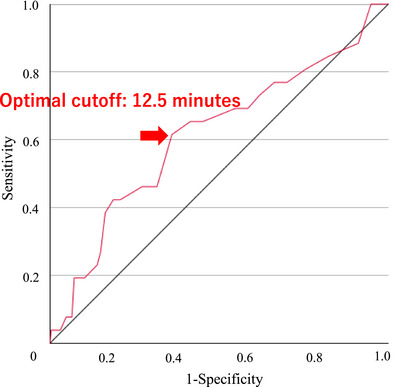
Receiver operating characteristic (ROC) curve for determining the optimal timing of precut sphincterotomy. The ROC curve illustrates the predictive performance of the timing of precut sphincterotomy for post‐ERCP pancreatitis (PEP). The area under the curve (AUC) was 0.613, indicating moderate discrimination. Based on ROC analysis, the optimal precut start time was determined to be 12.5 min, which yielded a sensitivity of 61.5% and specificity of 63.9%. This threshold attained the highest Youden Index (0.255), representing the best balance between sensitivity and specificity for PEP prediction.

An ROC analysis was performed to determine the optimal timing for precut sphincterotomy to prevent PEP. The area under the curve was 0.613, indicating moderate predictive accuracy (Figure 2). The optimal cutoff value for precut timing was 12.5 min, with a sensitivity of 61.5% and specificity of 63.9%. This threshold revealed the highest Youden Index of 0.255, which indicates balanced performance in predicting PEP risk.

### Comparison between the optimized precut group and the delayed group

Based on the ROC‐determined cutoff value, patients were divided into two groups: the optimized precut group (≤12 min, *n* = 198) and the delayed group (> 12 min, *n* = 122; Table 2). The median precut start time was 5.0 ± 3.3 min in the optimized precut group and 16.0 ± 6.0 min in the delayed group, and the difference had borderline statistical significance (*p* = 0.05). Statistically significant differences were observed between the two groups in terms of operator experience (*p* < 0.01), presence of diverticula (*p* = 0.03), cannulation time (*p* < 0.01), and total procedure duration (*p* < 0.01; Table 2). The incidence of PEP was significantly lower in the optimized precut group than in the delayed precut group (5.1% vs. 13.1%, *p* = 0.02). There was no significant difference in the severity of PEP between the two groups (*p* = 1.00). Additionally, there were no significant differences in the incidence of post‐ERCP bleeding or perforation between the early and delayed precut groups.

**TABLE 2:** Characteristics of patients undergoing optimized and delayed precut.

	Optimized precut group (*n* = 198)	Delayed precut group (*n* = 122)	*p*‐value
Age	76.0 ± 14.4	77.0 ± 12.2	0.34
Gender, male	110 (55.6%)	59 (48.4%)	0.25
Anatomy			0.28
Normal	191 (96.5%)	114 (93.4%)	
Billroth I	7 (3.5%)	8 (6.6%)	
Diagnosis			0.54
Benign	138 (69.7%)	81 (66.4%)	
Malignancy	60 (30.3%)	41 (33.6%)	
First endoscopist			<0.01
Trainee	66 (33.3%)	95 (77.9%)	
Trainer	132 (66.7%)	27 (22.1%)	
Diverticular	42 (21.2%)	40 (32.8%)	0.03
Precut start time	5.0 ± 3.3	16.0 ± 6.0	<0.01
Cannulation time	12.0 ± 10.1	25.0 ± 12.2	<0.01
Success of cannulation	191 (96.5%)	113 (92.6%)	0.19
Additional endoscopic papillary procedures (not mutually exclusive)			
Non	70 (35.4%)	67 (54.9%)	<0.01
EST	98 (49.5%)	51 (41.8%)	0.21
EPBD	27 (13.6%)	2 (1.6%)	<0.01
EPLBD	16 (8.1%)	6 (4.9%)	0.40
EPS	92 (46.5%)	58 (47.5%)	0.91
Total procedure time	34.5 ± 16.1	43.5 ± 13.3	<0.01
Incident rate of PEP	10 (5.1%)	16 (13.1%)	0.02
Severity of PEP			1.00
Mild	7 (3.5%)	12 (9.8%)	
Moderate	2 (1.0%)	4 (3.3%)	
Severe	1 (0.5%)	0 (0%)	
Fatal	0 (0%)	0 (0%)	
Post‐ERCP bleeding	6 (3.0%)	3 (2.5%)	1.00
Perforation	0 (0%)	0 (0%)	1.00

Abbreviations: EPBD, endoscopic papillary balloon dilation; EPLBD, endoscopic papillary large balloon dilation; EPS, endoscopic pancreatic stenting; ERCP, endoscopic retrograde cholangiopancreatography; EST, endoscopic sphincterotomy; PEP, post‐endoscopic retrograde cholangiopancreatography pancreatitis.

### Multivariate analysis of risk factors for PEP

A multivariate binary logistic regression analysis was performed to identify independent risk factors for PEP (Table 3). The study revealed that delayed precut timing was a significant risk factor for PEP (OR = 3.134, 95% CI: 1.053–9.324, *p* = 0.04), whereas the use of EPS (OR = 0.284, 95% CI: 0.107–0.755, *p* = 0.01) significantly reduced the risk of PEP.

**TABLE 3:** Multivariable binary logistic regression to predict post‐endoscopic retrograde cholangiopancreatography pancreatitis (PEP) in precut patients.

Variable	OR	95% CI	*p*‐value
Age	0.991	0.958–1.025	0.602
Gender (Male)	0.727	0.310–1.705	0.464
Diverticular	1.175	0.445–3.106	0.744
First Operator (Trainer)	1.035	0.395–2.709	0.945
Optimized precut	3.134	1.053–9.324	0.04
Cannulation time	0.996	0.955–1.038	0.843
EPS	0.284	0.107–0.755	0.012
Total procedure time	1.004	0.973–1.037	0.781
Constant	0.153	–	0.172

Abbreviations: CI, confidence interval; EPS, endoscopic pancreatic stenting; OR, odds ratio; PEP, post‐endoscopic retrograde cholangiopancreatography pancreatitis.

## DISCUSSION

This study revealed an association between the timing of precut sphincterotomy and the incidence of PEP in difficult cannulation cases. The results demonstrated that conversion to precut sphincterotomy within 12.5 min could significantly reduce the incidence rate of PEP compared with a delayed precut. Multivariate analysis identified delayed precut timing and absence of EPS as independent risk factors for PEP. These results reveal the clinical importance of appropriate timing for precut sphincterotomy and the efficacy of EPS in preventing PEP, even in precut cases.

The present study showed that 12.5 min was the optimal time for conversion to precut sphincterotomy, based on our ROC analysis. This result provides clinical criteria for the conversion to precut sphincterotomy in difficult cannulation cases. Although previous studies have suggested that early precut may effectively reduce the risk of PEP, the optimal timing for transitioning to precutting remains unclear. Early precut is widely defined as 5–20 min.^13^, ^14^, ^15^ In this context, our study contributes to the operators by establishing a data‐driven threshold of 12.5 min, which may serve as a practical guideline for clinical decisions. Early precutting prevents PEP by avoiding excessive manipulation of the major papilla. Prolonged cannulation attempts have been associated with an increased risk of PEP because repeated mechanical and chemical stimulation can lead to inflammation and pancreatic duct injury.^16^, ^17^

The results of the ROC curve analysis in this study suggested that conversion to precut at 12.5 min provided an appropriate balance between avoiding delayed and premature precut sphincterotomies, which may expose patients to an increased risk of other adverse events. Converting to precut sphincterotomy at this threshold may reduce the risks associated with prolonged cannulation, while still allowing sufficient time for conventional cannulation attempts. However, early conversion to precut carries a risk of other complications. For example, if a precut is performed prematurely, patients who could have been successfully cannulated using conventional methods may undergo unnecessary precut sphincterotomy, which can lead to an increased risk of complications such as bleeding and perforation. Compared with standard cannulation techniques, precut sphincterotomy inherently involves greater technical difficulty and carries a higher risk of adverse events.^18^ Thus, precut should not be performed indiscriminately but at an optimal time that balances the benefits of reducing PEP risk with the risks of procedural complications. In our study, biliary cannulation was ultimately successful in 304/320 (95.0%) of cases, suggesting that early precut can be safely implemented in appropriate clinical settings without compromising procedural outcomes.

In terms of education, endoscopists must develop technical skills for conventional cannulation and clinical assessment to determine when conversion to precut sphincterotomy is appropriate. Overemphasis on early precut could lead to an awareness of indiscriminate precut sphincterotomy and an increase in severe complications. This approach can improve technical skills and clinical judgment, minimize avoidable complications, and ultimately improve patient safety and procedural outcomes. Furthermore, by adhering to an objective threshold such as 12.5 min, training programs can provide a structured approach to precut decision‐making, reduce variability in practice, and improve standardization.

Second, EPS reduced the risk of PEP, even in precut sphincterotomy cases in the present study, and the use of EPS is widely recognized as an effective method for preventing PEP. Inadvertent pancreatic duct cannulation, contrast injection, or mechanical manipulation can lead to temporary obstruction of the duct, causing pancreatic fluid stasis and ductal hypertension, which contribute to the development of PEP.^19^ One of the main preventive mechanisms of EPS is decompression of pancreatic intraductal pressure by facilitating pancreatic juice drainage. Previous studies have demonstrated that EPS significantly lowers the incidence of PEP, particularly in high‐risk cases, such as those involving multiple pancreatic duct cannulations or precut sphincterotomy.^8^, ^19^, ^20^ At our institution, EPS is commonly performed at the end of ERCP, particularly in patients who are difficult to cannulate. However, some reports have suggested that performing EPS before precut sphincterotomy may improve the success rate of bile duct cannulation.^21^, ^22^ One proposed mechanism is that securing the pancreatic duct with a stent may reduce the involuntary movement of the papilla, thereby allowing for a more controlled and effective precut sphincterotomy. This approach also has the potential to improve the success rate of bile duct cannulation in addition to preventing PEP. Thus, EPS is a valuable technique for preventing PEP in precut cases and potentially improving the success rate of precut sphincterotomy.

This study has certain limitations that should be noted when interpreting our findings. First, this study was retrospective and conducted at a single center. Second, the definition of precut timing and its applicability in various clinical settings may differ, thus necessitating external validation. Additionally, the decision to perform EPS was at the discretion of the endoscopist, which may have introduced variability. Finally, the study included a variety of endoscopists whose differences in technical skills may have affected the incidence of PEP. A limitation of this study is the differing strategies between trainees and experienced endoscopists. Trainees more often used alternative techniques before precut, contributing to longer procedures. Although operator experience was not an independent risk factor for PEP in multivariate analysis, this procedural difference may have influenced outcomes. Future studies should be stratified by operator experience.

In conclusion, the findings of this study suggest that conversion to precut sphincterotomy within 12.5 min and performing EPS are effective factors in reducing the incidence of PEP. These findings underscore the importance of timely decision‐making during ERCP and highlight the need for proactive PEP prevention strategies in high‐risk patients.

## CONFLICT OF INTEREST STATEMENT

None.

## ETHICS STATEMENT

The study protocol was approved by the Institutional Research Ethics Committee (Approval No: 5984‐01). Animal studies were not applicable.

## PATIENT CONSENT STATEMENT

This study was conducted with informed consent, in the form of an opt‐out on the website.

## CLINICAL TRIAL REGISTRATION

N/A.
